# Cannabis-Based Phytocannabinoids: Overview, Mechanism of Action, Therapeutic Application, Production, and Affecting Environmental Factors

**DOI:** 10.3390/ijms252011258

**Published:** 2024-10-19

**Authors:** Marta Jurga, Anna Jurga, Kacper Jurga, Bartosz Kaźmierczak, Katarzyna Kuśmierczyk, Mariusz Chabowski

**Affiliations:** 1 4th Military Teaching Hospital, 50-981 Wroclaw, Poland; marta.jurga9@gmail.com (M.J.); kacper.jurga123@gmail.com (K.J.); 2 Faculty of Environmental Engineering, Wroclaw University of Science and Technology, Wybrzeże Wyspiańskiego 27, 50-370 Wroclaw, Poland; anna.jurga@pwr.edu.pl (A.J.); bartosz.kazmierczak@pwr.edu.pl (B.K.); 3 District Medical Center in Grójec, Piotra Skargi 10, 05-600 Grójec, Poland; kusmieczyk.kasia@gmail.com; 4 Faculty of Medicine, Wroclaw University of Science and Technology, Hoene-Wrońskiego 13c, 58-376 Wroclaw, Poland

**Keywords:** phytocannabinoids, medical cannabis, therapeutic use, CBD, indoor cultivation

## Abstract

This review provides an overview of cannabis-based phytocannabinoids, focusing on their mechanisms of action, therapeutic applications, and production processes, along with the environmental factors that affect their quality and efficacy. Phytocannabinoids such as THC (∆^9^-tetrahydrocannabinol), CBD (cannabidiol), CBG (cannabigerol), CBN (cannabinol), and CBC (cannabichromene) exhibit significant therapeutic potential in treating various physical and mental health conditions, including chronic pain, epilepsy, neurodegenerative diseases, skin disorders, and anxiety. The cultivation of cannabis plays a crucial role in determining cannabinoid profiles, with indoor cultivation offering more control and consistency than outdoor methods. Environmental factors such as light, water, temperature, humidity, nutrient management, CO_2_, and the drying method used are key to optimizing cannabinoid content in inflorescences. This review outlines the need for broader data transfer between the health industry and technological production, especially in terms of what concentration and cannabinoid ratios are effective in treatment. Such data transfer would provide cultivators with information on what environmental parameters should be manipulated to obtain the required final product.

## 1. Introduction

Cannabinoids called phytocannabinoids originate from plants, including *Cannabis sativa* (hemp), and have potent therapeutic potential in treating various medical conditions [1]. Hemp has been used around the world as a source of food and textile fiber since ancient times, with further recognition of its medicinal properties [2]. Since the 19th century, disorders such as epilepsy, migraine, asthma, neuralgia, fatigue, and insomnia have been treated using cannabis, with phytocannabinoids playing a vital role [3]. The human brain responds to phytocannabinoids through cannabinoid receptors (CBx), which are part of the endocannabinoid system [2]. The endocannabinoid system, made up of the enzymes involved in the production and breakdown of endocannabinoids, cannabinoid receptors (CB1 and CB2), and endogenous ligands (endocannabinoids), is essential for preserving homeostasis in several physiological processes. This correlation allows the compounds to affect metabolic, immune, nervous, energy balance, sleep, memory, or mood functions.

Phytocannabinoid content covers nearly 25% of all chemicals identified in cannabis composition. Until now, more than 120 have been classified with the potential to discover more given the increasing popularity of this field in the last few decades [1]. The most extensively studied phytocannabinoids are cannabidiol (CBD) and ∆^9^-tetrahydrocannabinol (∆^9^-THC). The first is a non-psychoactive compound exhibiting potential anti-inflammatory, neuroprotective, and anxiolytic properties. ∆^9^-THC is known for its psychoactive effects and for possessing analgesic and antiemetic properties. A wide range of additional phytocannabinoids have shown intriguing pharmacological effects, including cannabichromene (CBC), cannabigerol (CBG), and cannabinol (CBN).

Beyond their effects on the endocannabinoid system, phytocannabinoids are studied for their ability to modify ion channels, neurotransmitter receptors, and antioxidative pathways. The complex interaction between phytocannabinoids and biological systems offers hope for novel treatment approaches and lays the groundwork for further developments in cannabinoid-based medicine [4].

The subject of cannabis cultivation and cannabinoid extraction has been gaining tremendous popularity in recent years, bringing together many interdisciplinary areas of knowledge. On one hand, detailed research is being conducted on the effects of cannabinoids in the treatment of various medical conditions, while on the other hand, technologies related to the cultivation of hemp are being developed, which have a key impact on the quality and composition of the final products. Analyzing how different cultivation parameters and technological processes affect the cannabinoid content of the final product provides a better understanding of its therapeutic potential. It is worth noting that differences in cannabinoid composition, which can be as high as 25% between products of the same cannabis strain, underscore the need for further research into how environmental and process factors affect the quality of the final product [5]. The integration of these two fields—the medical properties of cannabinoids and the technology of their cultivation and production—is essential to optimize production methods and ensure the high quality and effectiveness of hemp products.

This review focuses on the combination of two areas related to bioactive cannabinoids: medical and technological. On one hand, the therapeutic potential of various cannabinoids, their mechanism of action, and the form of preparations used in the health system are presented. On the other hand, from the technological side, we present the parameters throughout the life cycle of a cannabinoid product that can be manipulated to achieve the desired end formulation.

## 2. Cannabis-Based Phytocannabinoids Overview

### 2.1. The Endocannabinoid System (ECS)

Biological effects of phytocannabinoids on humans are related to the neuromodulation of the endocannabinoid system (ECS) by interacting with various receptors [6]. The ECS can be divided into three main parts. The first part consists of the two 7-transmembrane-domain, G protein-coupled receptors: cannabinoid receptor type-1 (CB1) and cannabinoid receptor type-2 (CB2). The second part includes the endogenous ligands (endocannabinoids). The third part comprises the enzymes responsible for endocannabinoid metabolism [7]. The ECS system is involved in maintaining homeostasis in the human body. It is manifested by its impact on various systems, from maintaining proper body temperature through influencing mood to strengthening the immune system. However, ECS affects much more than just physiological processes. Among the areas it influences are anxiety, feeding behavior/appetite, emotional behavior, depression, nervous functions, neurogenesis, neuroprotection, reward, cognition, learning, memory, pain sensation, fertility, pregnancy, and pre- and post-natal development. It has also been observed that there is some influence on the pathophysiological development of certain diseases, including tumors, cardiovascular diseases, and neurodegenerative diseases [8]. As a result, it is receiving increasing attention from the scientific community.

### 2.2. Tetrahydrocannabinol (∆^9^-THC)

THC shows a wide range of effects, which are outlined in Table 1. The table illustrates how THC operates through specific receptors and the resulting biological responses it triggers. Tetrahydrocannabinol (∆^9^-THC) is a major active substance present in *Cannabis sativa* [9]. It interacts with the ECS by binding to CBR1 and CBR2 receptors [10]. Its acidic precursor is THCA-A (∆^9^-tetrahydrocannabinolic acid A) [9]. THCA-A is also stored in glandular trichomes of flowers and leaves, which constitutes the main part of total THC in *Cannabis sativa* [11]. Furthermore, as the plant becomes mature, it acts as a necrosis-inducing factor. As THCA-A is stored and fermented, it forms THC through the decarboxylation process. If it were further exposed to temperature and light, it would transform into cannabinol [12]. Non-enzymatic decarboxylation can occur during the smoking of plant material [13]. Nevertheless, the entire compound does not undergo the process of decarboxylation. That is why it can be found in the bodily fluids of a person who smokes *Cannabis sativa*. It is considered a marker, which can show if the person had used synthetic THC or cannabis [12].

The effects of THC are observed through its broad effects on various receptors. THC ranges from acting as a partial agonist for the CBR1 and CBR2 receptors to being a full agonist for the GPR55/GPR18, TRPV2-4/TRPA1, PPARa/y receptors to being an antagonist for the TRPM8/5HT3A receptor [15].

THC administered occasionally causes increased neuronal activity by increasing the amount of dopamine. However, long-term exposure to THC causes reduced dopamine concentration and, consequently, reduced neuronal activity [26].

The effect of THC on serotonin can also be observed. At higher THC concentrations, the inhibition of serotonin uptake was observed. Nevertheless, with chronic THC abuse, the maximum speed of serotonin uptake increases [27].

### 2.3. Cannabidiol (CBD)

Unlike THC, Cannabidiol (CBD) does not have psychoactive effects but still affects the ECS. This manifests itself in the influence on various types of receptors, as shown in Table 2, which also details the corresponding effects. Among the wide range of effects on various receptors, the most noteworthy are the CB1 and CB2 receptors. CBD acts as a negative allosteric modulator of the CB1 receptor. It has an agonist effect on the CB2 receptor. CDB also has an agonistic effect on 5-HT1A and α1-Adrenergic α1-Adrenergic. CBD is an inhibitor of GPR55 receptors as well as TRPM8 channels. However, it is an activator of PPARγ receptors and TRP channels [28].

### 2.4. Cannabigerol (CBG)

Cannabigerol (CBG) is a phytocannabinoid that, similarly to the previous one, has no psychotropic properties. It occurs in cannabis in trace amounts. Its precursor is cannabigerolic acid. CBG acts as a partial agonist of the CB1 and CB2 cannabinoid receptors. But it also affects other receptors, such as TRPV1, TRPV2, TRPA1, and TRPM8 channels. Moreover, the activation of α2-adrenergic receptors by CBG in the kidneys and the blocking of 5-HT1A receptors by this compound in the brain were also observed. The latest research shows that CBG has an appetite-stimulating effect in rats. It is also important that no negative effects were observed after such administration. Additionally, the latest research shows that CBG has an appetite-stimulating effect in rats. It is also important that no negative effects were observed after such administration. Moreover, CBG is postulated to have antiepileptic effects [34].

### 2.5. Cannabinol (CBN)

Cannabinol (CBN) is produced as a result of the oxidative breakdown of Δ^9^-THC during aging or exposure to light. It has weak psychoactive effects. It binds to CB1 and CB2 receptors, towards which it has a partial agonist effect. CBN has also been shown to affect other ECS receptors. CBN is an agonist of the TRPA1 channel and also an antagonist of the TRPM8 channel [35].

### 2.6. Cannabichromene (CBC)

Cannabichromene (CBC) is a phytocannabinoid that does not have psychoactive properties. Although it is one of the less important cannabinoids found in cannabis, it still has a number of potential health and therapeutic benefits. This is mainly related to its effect on TRPA1 channels. CBC is the strongest agonist of TRPA1 channels [24]. However, when it comes to CB1 and CB2 receptors, it has low affinity towards them. Therefore, its biological effects are not related to CB1 and CB2 receptors.

Among other channels influenced by CBC, we can also distinguish TRPV3 and TRPV4 channels, which are also activated by CBC. However, TRPM8 channels are inhibited. It is worth emphasizing that the effect on these channels is not as strong as on TRPA1 channels.

## 3. Phytocannabinoids Therapeutic Potential in Physical and Mental Health Conditions Treatment

Phytocannabinoids, due to their multifunctional mechanism of action on the human body, show many potential applications in medicine [36,37]. They demonstrate a beneficial effect on many disease states, both somatic and mental. Somatic disease states in which phytocannabinoids can be used include pain of various types [12], many neurodegenerative diseases [38], epilepsy [39,40], cancer [41], rheumatic diseases [42], skin diseases [43], gastrointestinal diseases [44], nausea [45] and many others [46]. When it comes to mental illnesses, phytocannabinoids could be used in diseases such as depression, anxiety, and sleep disorders [47,48]. The following section of the article describes several important potential applications of individual phytocannabinoids in somatic and mental diseases, which are important from epidemiological and civilizational points of view.

### 3.1. ∆^9^-THC

#### 3.1.1. Pain Management

THC’s well-known therapeutic potential is to reduce chronic pain [49,50]. The current standard of treatment for chronic pain involves opioid analgesics, which have a lot of severe side effects, including nausea, sedation, opioid-induced hyperalgesia, depression of the respiratory center, and opioid dependence. Due to that, cannabinoids may replace opioids in the future in pain management [49,50,51]. THC induces analgesia by interacting with at least three targets: CB1, CB2, and Gly receptors [49]. The CB1 receptor is, however, the target that THC activates with the highest potency; CB1 receptors are abundant in nociceptive and non-nociceptive sensory neurons of the dorsal root ganglion (DRG), the spinal cord, the brain, mast cells, macrophage defense cells, and the trigeminal ganglion (TG) [49,51]. The CB1 receptor is responsible for THC’s psychotropic effects [6]. THC activates the CB1 receptor as a partial agonist and modulates neuronal functions by reducing presynaptic neurotransmitter release (e.g., glutamate, GABA, and acetylcholine). CB2 receptors are not highly expressed in these regions; however, they are increased when there is peripheral nerve damage [51]. The activation of the CB2 receptor reduces inflammation pain [50].

Studies show that the acute inhalation of THC (0.5–1 mg) produces greater analgesia compared to the placebo in chronic pain patients and causes only slight side effects that resolve spontaneously [49,52]. THC can reduce pain by acting in different ways; it has the ability to inhibit prostaglandin E-2 synthesis, increase cerebral production of 5-hydroxytryptamine (5-HT), alter dopaminergic function, and inhibit pre-synaptically glutamate release [51]. The analgesic effect of THC has been well described in rodent studies for the treatment of pathological and trauma-related pain [49,53]. THC also showed a positive effect on patients with fibromyalgia, with both symptom and pain relief observed [42,54].

On the other hand, it is worth pointing out that adverse events of cannabis, such as fatigue, tachycardia, and dizziness, are mainly attributed to THC; therefore, the total daily dose equivalent of THC should generally be limited to 30 mg/day or less, preferably combined with CBD [51]. Different doses and routes of administration of CBD have different effects on THC’s bioactivity [49].

#### 3.1.2. Seizures

THC (30 mg/kg i.p.) reduces chemically induced seizures in rats through a CB1 receptor-dependent mechanism [49,55]. THC and CB1 receptor agonists reduce excitatory transmission and hippocampal overexcitation known to cause seizures; however, due to the side effects of THC, studies rather moved to target the eCB signaling and the allosteric site of CB1 receptor [49]. There are, however, new studies that may suggest the usefulness of THC in the treatment of treatment-resistant epilepsy in children [39].

#### 3.1.3. Neurodegenerative Diseases

The potential of THC in treating neurodegenerative diseases may be great. Scientific reports suggest that THC may find application in treating diseases such as Parkinson’s disease, Alzheimer’s disease, and multiple sclerosis [29,38,46,56]. These are particularly important diseases due to the increasing number of new cases each year, as well as their possible severe clinical course [57]. There are also isolated reports on the effect of THC on other neurodegenerative diseases, such as amyotrophic lateral sclerosis, but they are not described below due to the small amount of research and scientific evidence [46]. The potential use of THC in the most common neurodegenerative diseases mentioned earlier is described in more detail below.

- Parkinson’s disease

THC showed neuroprotective effects in preclinical studies. It had reduced cell death response after induction with toxins designed to induce oxidative stress, such as paraquat [58]. In rodent studies, in a Parkinson’s disease model, THC administered intraperitoneally showed the potential to increase dopamine levels in the substantia nigra [59]. In clinical trials, however, the effects of THC have not yet been clearly described [60]. For example, in the study examining the use of THC and CBD in Parkinson’s disease patients who received THC/CBD treatment, the results showed reduced anxiety, pain, and improved sleep in PD patients [61]. However, these individuals also showed increased non-motor symptoms and cognitive difficulties. Further clinical studies on a large population are required to more accurately assess the effects of THC on Parkinson’s disease in humans.

- Multiple Sclerosis

There are studies that revealed that cannabis may also slow the neurodegenerative processes in multiple sclerosis [62]. The administration of an oromucosal spray that contains THC and CBD in a 1:1 ratio led to a decrease in spasticity and pain relief in patients with MS, according to the results of clinical research [38,63].

- Alzheimer’s Disease

Some studies showed that THC demonstrated anti-amyloid aggregation activity, promoted the destruction of intracellular Aβ, inhibited the inflammatory reaction, and blocked acetylcholinesterase activity [38]. Cognitive functions in old mice improved after the treatment with a low dose of THC [64]. Further research is required to confirm the positive effect of THC on Alzheimer’s disease patients.

#### 3.1.4. Nausea

The addition of oral THC:CBD to standard antiemetics was associated with less nausea and vomiting in patients with chemotherapy treatment [45].

#### 3.1.5. Taste Alteration/Appetite

Patients with IBD (inflammatory bowel disease) are in a high-risk group of poor nutrition and malnutrition [44]. A study was conducted that examined the effects of medical cannabis on appetite in patients with IBD [44]. Medical cannabis for patients who were treated to boost their appetite contained higher concentrations of THC. Results showed that a third of the patients following such therapy reported a significant increase in their appetite after 3 months, with a modest increase in BMI after 6 months.

#### 3.1.6. Sleep Quality

THC may have a dose-dependent effect on sleep; low doses are believed to reduce sleep onset latency and increase slow-wave sleep and total sleep time; however, high doses tend to cause sleep disturbances and increase insomnia symptoms [65]. Orally administered THC showed potential in treating sleep disturbances in people suffering from post-traumatic stress disorder [66]. Effects of two different soft gel dietary supplements were studied, one with lower THC and higher levels of other botanicals, and the other one with higher THC and lower levels of other botanicals, on sleep disturbance in comparison to placebo [67]. These two formulations contained the same amount of CBD, CBN, and L-theanine. The first set contained lower amounts of THC (0.35 mg) and higher amounts of GABA (150 mg), hops oil (75 mg), and valerian oil (75 mg). The second formula contained 0.85 mg THC, 125 mg GABA, 20 mg hops oil, and 20 mg valerian oil. After taking those kits for 4 weeks, the participants were asked to complete online surveys of their sleep, containing issues on the subject of their feelings of anxiety, stress, pain, and overall well-being. The results showed that there was a significant difference in effect between the first formula and placebo control; however, there was no significant difference in effect on any health outcomes between the second formula and placebo control. It shows that the strains with lower THC may be more effective at promoting sleep, which confirms the dose-dependent effect [68].

It is important to remember that the research base confirming the positive effect of THC and cannabinoids in general on sleep quality is still very small, but looking at the promising results of individual studies, it is necessary to devote more time to this topic in the future [69].

#### 3.1.7. Anxiety

It is worth pointing out that cannabis that is rich in THC induces anxiety, but low doses are potentially anxiolytic [65,67].

### 3.2. CBD

#### 3.2.1. Pain Management

CBD has been proven to be safe and causes only mild adverse effects in humans such as ataxia, sedation, nausea, headache, or decreased appetite [70]. CBD is believed to control pain through different mechanisms; for example, by interacting with vanilloid-transient receptor potential-1 (TRPV-1) or the capsaicin receptor as an agonist, which results in inhibiting the fatty-acid amide hydrolase enzyme (FAAH), which is responsible for the hydrolysis of anandamide and inhibits its reuptake. Anandamide is an endogenous cannabinoid that shows affinity for CB1 and CB2 receptors. CBD may also improve anti-inflammatory effects by decreasing reactive oxygen species (ROS), tumor necrosis factor (TNF-α) levels, and pro-inflammatory cytokines [51].

Several studies have reported that in healthy rodents subjected to a painful experience, the administration of CBD may diminish the nociceptive experience. In a sciatic nerve injury mouse model, the administration of CBD-containing gelatine significantly reduced allodynia up to 3 weeks post-surgery. In rats, after ligation of the L5 spinal nerve, CBD suppressed chronic neuropathic pain. CBD may also have an influence on pain reduction in inflammatory and arthritis-related pain [70].

In humans, CBD (300 mg/oral/daily) prevents acute and transient chemotherapy-induced peripheral neuropathy [49]. A transdermal CBD-containing gel in patients with peripheral neuropathic pain caused pain alleviation, as well as the alleviation of cold and itchy sensations; however, a majority of clinical studies describe the efficacy of CBD and Δ^9^-THC co-administration [70]. To better assess the usefulness of CBD in pain management, further human studies are required.

#### 3.2.2. Epilepsy

The available meta-analyses indicate that CBD is the most effective cannabinoid, reducing seizure frequency (SF) in both experimental and clinical conditions [40]. CBD may be effective in patients with treatment-resistant epilepsy, especially in patients with Dravet and Lennox–Gastaut syndrome, as an adjunctive therapy to their current anti-epileptic medications [40,49]. Some studies showed that additional doses of oral CBD for 3 months may, in some cases, reduce weekly seizure frequency by almost 50% [46].

#### 3.2.3. Skeletal Muscle Regeneration

CBD may affect muscle regeneration. The study examined the effect of CBD supplementation on Skeletal Muscle Regeneration after Intensive Resistance Training [71]. Research results showed that participants who took CBD had lower blood levels of markers of muscle damage such as creatine kinase and myoglobin 72 h after training [71]. Additionally, this group was able to return to their maximum strength levels faster than the placebo group.

#### 3.2.4. Neurodegenerative Diseases

Due to its antioxidant and anti-inflammatory effects, CBD may have a positive impact on many neurodegenerative diseases [3,56,72]. This work, however, focuses on the use of CBD in the most common ones, on which the most research has been conducted. We describe the potential use of CBD in Parkinson’s disease, Alzheimer’s disease, and multiple sclerosis as follows. There are also individual reports of a positive effect of CBD on amyotrophic lateral sclerosis and Huntington’s disease, but due to the small number of studies and unclear conclusions from these studies, these issues were not described in more detail in this paper [46].

- Parkinson’s disease

CBD may improve the functioning of people with Parkinson’s disease [29]. It was reported that CBD reduced psychotic symptoms; however, the number of clinical trials remains limited [38,73]. A small study by Chagas et al. on people with Parkinson’s disease provides preliminary evidence for the positive effects of CBD on this disease. Patients were given CBD orally for 6 weeks. The results showed that CBD improved the overall quality of life in these patients, as well as having a positive effect on the quality of sleep in PD patients with REM sleep behavior disorder [72,74].

- Multiple Sclerosis

Preclinical studies show that thanks to CBD’s immunomodulatory effect, it may inhibit the onset and progression of multiple sclerosis [38]. In rodent studies in a multiple sclerosis model, desirable effects were obtained after the simultaneous administration of CBD and THC compared to these substances administered separately [75]. Some human studies confirm the positive effect of combining CBD and THC in reducing spasticity in people with multiple sclerosis [3,63,76]. Their potential may also be related to reducing pain associated with multiple sclerosis, but further clinical trials on large populations are also required in this regard [63,75].

- Alzheimer’s disease

CBD may also have an influence on the course of Alzheimer’s disease [1,32,72]. Studies conducted on rodents show that CBD is able to reduce inflammation caused by the neurotoxicity of amyloid beta peptide (Aβ) and increase the survival and neurogenesis of the rat and mouse hippocampus [32]. A 2022 study on a mouse model of Alzheimer’s disease showed that the intraperitoneal administration of CBD had a positive effect on spatial memory and reduced anxiety-like behaviors [72,77]. Further research is required to further evaluate the effects of CBD on Alzheimer’s disease in humans.

#### 3.2.5. Skin Diseases

All the classes of cannabinoids interact with cannabinoid receptors located in the skin and regulate the pathways, which affect the metabolism of skin appendages and cutaneous cells [78]. Cannabinoids may affect the skin on many levels; they can influence hormone secretion by interacting with cell receptors or indirectly, by influencing stress responses [79]. Studies show that due to CBD’s antioxidant and modulating effects on the endocannabinoid system, it can rescue keratinocytes and melanocytes from UV-B-induced cytotoxicity [43]. CBD showed the highest activity to inhibit keratinocyte proliferation among Δ^9^-THC, CBD, CBG, and CBN [26]. It is worth pointing out that in patients with psoriasis, when there is an increase in oxidative stress in granulocytes and serum, CBD tends to increase the oxidative status, which may suggest its potential use as an antioxidant [43,79,80]. Another impact of CBD, which has an anti-inflammatory effect, suggests its usefulness in acne treatment [81,82]. Studies show that CBD was able to inhibit excessive lipid synthesis (lipogenesis) in sebocyte cultures and reduce concentrations of inflammatory cytokine TNF-α [81,82].

#### 3.2.6. Liver Function/Insulin Resistance

The endocannabinoid system in animal models of obesity, and in selected studies of humans seems to be upregulated, and it is believed that may contribute to an unfavorable metabolic phenotype [83]. Abbotts et al. conducted a study that showed that taking CBD after a meal may have a beneficial effect on the insulin and triglyceride response [83].

#### 3.2.7. Taste Alteration/Appetite

Taste alteration is a common adverse effect of chemotherapy [84]. A study that examined the effects of CBD on the prevention of taste alterations in patients with cancer was conducted [84]. Results showed that patients receiving CBD had a better ability to differentiate between strong and weak tastes after three cycles of chemotherapy compared with the placebo group.

#### 3.2.8. Sleep Quality

Other authors examined cannabidiol supplementation on sleep quality [85]. The results showed that daily ingestion of 50 mg CBD, 1–1.5 h before sleep onset, leads to significantly improved perceived sleep quality compared with a placebo group. The potential mechanism of action, in this case, is likely related to the activation of CB1 receptors, which can be found in brain regions involved in the sleep–wake cycle. Their activation is believed to cause an increase in the amount of slow-wave and REM sleep.

Other studies researched the influence of cannabidiol on sleep quality [86]. Participants with sleep disturbances took orally ingested CBD, alone or with additional CBN for 4 weeks. Results confirm the positive impact of CBD on reducing sleep disturbances; however, these effects do not exceed that of 5 mg melatonin. It is worth noting that additional doses of minor cannabinoids like CBN did not impact the therapeutic effects of CBD. The above results suggest that sleep disorders may be a niche in which CBD will find wide usefulness in the future.

#### 3.2.9. Anxiety

Single studies show that CBD may have anti-anxiety effects [49,87,88]. Anxiolytic effects are believed to be caused by activating different mechanisms of action depending on the dose [87]. Acute anxiolytic effects of CBD at low and intermediate doses are believed to be caused by integrating with 5-HT1A receptor activation, and higher CBD doses are thought to involve TRPV1 receptor antagonism action. Preliminary results suggest the effectiveness of CBD in reducing anxiety in people with social anxiety disorder and PTSD [87,88].

#### 3.2.10. Psychosis

There is some evidence suggesting that CBD may attenuate THC-induced paranoid symptoms [65,68]. Additionally, single studies showed positive effects of CBD as a monotherapy in patients with schizophrenia. The effect of reducing positive and negative symptoms was comparable to antipsychotic drugs [89]. It is important to remember that further research is needed to better assess the usefulness of CBD in treating psychosis.

### 3.3. CBG

#### 3.3.1. Pain Management

Recent research in mice with fractured limbs suggests that CBG has the potential to attenuate post-fracture pain efficiently and promote bone healing [90]. Cisplatin-induced peripheral neuropathy (CIPN) may be another condition where CBG can be used [91]. Other mouse studies show that CBG efficiently reduces neuropathic pain in a mouse model of CIPN in male and female mice without the development of tolerance and without the need to increase the dose [91]. Probably because of its ability to act as partial agonistic effects on CB2 receptors, CBG may also have an anti-inflammatory effect, so it may have the potential to reduce pain associated with inflammation [92].

#### 3.3.2. Taste Alteration/Appetite

CBG may be helpful in the treatment of chemotherapy-induced cachexia [78,93]. Studies on rodents show that CBG has the potential to stimulate appetite in healthy rats without neuromotor side effects in an acute cachectic phenotype model induced by cisplatin and can reduce cisplatin-induced weight loss [78,93].

#### 3.3.3. Skin Diseases

Studies show that CBG may have a dose-dependent effect to inhibit inflammatory cytokines such as IL-1β, IL-6, IL-8, and TNF-α in response to inflammatory inducers such as ultraviolet light and sunlight but also angiogenic growth factors [78,94]. In addition, compared to CBD, CBG exhibits twice the antioxidant activity but has a slightly smaller effect when it comes to the inhibition of keratinocyte proliferation [79]. A study using human skin equivalents demonstrated that CBG effectively reduces reactive oxygen species (ROS) in human dermal fibroblasts, being nearly 1800 times more effective than ascorbic acid (vitamin C) in this regard [80,94]. Thanks to CBG’s anti-inflammatory, antioxidant, and antimicrobial effects, it may have a significant role in Atopic Dermatitis treatment [79].

#### 3.3.4. Neurodegenerative Diseases

VCE-003, a derivative of CBG quinone, has also demonstrated neuroprotective activity in various experimental models of Huntington’s disease and Parkinson’s disease [94].

#### 3.3.5. Liver Function/Insulin Resistance

Bzdęga et al. conducted a study where the administration of CBG on sphingolipid deposition in the liver of insulin-resistant rats [95]. The rat model of insulin resistance was induced by a high-fat, high-sucrose diet. The results showed that CBG treatment may potentially prevent liver steatosis and may have a positive impact on insulin sensitivity by influencing the synthesis, degradation, and transport of sphingolipids in the liver.

### 3.4. CBN

#### 3.4.1. Skin Diseases

CBN has the ability to inhibit excessive keratinocyte proliferation, slightly lower than CBD but stronger than Δ^9^-THC and CBG, so it has great potential to be used in psoriasis treatment [79].

#### 3.4.2. Neurodegenerative Diseases

Porphytomonas gingivalis is an important bacterial pathogen associated with sporadic Alzheimer’s Disease [38]. CBN showed anti-inflammatory effects by suppressing P. gingivalis-induced release of pro-inflammatory cytokines such as IL-12 p40, IL-6, IL-8, and TNFα [38]. It can also have protective effects on mitochondria homeostasis, which may additionally confirm its usefulness in neurodegenerative diseases [96].

### 3.5. CBC

#### 3.5.1. Pain Management

CBC shows the ability to act as an agonist of the CB2 receptor, as well as the TRPA1, TRPV1, TRPV3, and TRPV4 ion channels [97]. It can also interact with CB1 and peroxisome proliferator-activated receptors (PPARs); some of these receptors are involved in pain management. Research conducted on mice by Raup-Konsavage et al. showed that CBC has broad pain control properties; it was able to reduce neuropathic, acute, inflammatory, and radiant heat pain [97].

#### 3.5.2. Skin Diseases

CBC also has the potential to inhibit the proliferative activity of keratocytes and has anti-inflammatory effects, which gives it the potential to be used in the treatment of psoriasis and acne [79].

### 3.6. Summary

As can be noted from the information presented above, the potential use of cannabinoids in the treatment of various conditions is extensive. A summary of the studies conducted, the therapeutic doses used, and their effect on the examined symptom is summarized in Table 3.

## 4. Major Production Process Parameters Affecting Phytocannabinoid Profile

In order to properly describe the technological production parameters that will affect the phytocannabinoid profile, it is necessary to distinguish between the various manufacturing steps throughout the process of obtaining the final cannabinoid product. For medicinal purposes, it may be in the form of dried inflorescences or in the form of a product based on an extracted blend of cannabinoids from the dried plant material (ex., in gel or oil form). The latter one, in addition to all the variables affecting the plant and its cannabidoid profile during cultivation, will be factors resulting from the selected post-harvest extraction method. In this study, we focus primarily on parameters influencing the quality of final dried inflorescences.

Thus, Section 4.2 describes the cultivation process and its influence on cannabinoid profile, which is common to each cannabinoid product, while Section 4.3 briefly discusses extraction methods, and their influence on the extracted cannabinoid content.

### 4.1. Place of Accumulation in Plant Material

Cannabis plants accumulate phytocannabinoids in glandular trichomes, which are distributed throughout the above-ground parts of the plant, with the highest concentrations found on female flowers (inflorescence) [104]. The harvested inflorescence is then given to dry and in this form can be used for therapeutic inhalation [105]. However, if another dosage form of cannabinoids is used (gel, oil, etc.), it is necessary to carry out an extraction process of the dried inflorescence [106].

### 4.2. Cultivation Process and Influence of Environmental Factors on Cannabinoid Content

*Cannabis sativa* is a wind-pollinated species that is highly allogamous in nature. It is a dioecious plant, with male and female flowers developed on separate plants when grown naturally from seed [107].

Female-only crops are preferred for cannabinoid production. Male plants produce much smaller amounts of cannabinoids, and pollinated females divert resources from cannabinoid production to seed development. To avoid this process, the removal of male plants is required based on their different flower structures. Cannabis is grown in fields, greenhouses, and specialized controlled facilities that create a closed environment [108,109,110].

Field cultivation provides several advantages, including ample space for root growth and unrestricted plant height, as well as access to free, full-spectrum sunlight essential for healthy plant development. However, the unpredictability of weather and seasonal changes makes it difficult to regulate light levels consistently, often forcing the use of supplementary lighting in greenhouses. Additionally, wind-spread spores and insects challenge disease control and pest infestations. Extreme weather conditions damage (such as floods, frost, hail, rain, wind, or extreme heat) poses further risks [111]. The potential for pesticide contamination is also heightened outdoors, either from drift or leaching from nearby fields or the persistent presence of banned pesticide residues applied in the past.

Thus, field cultivation is associated with significant inter-plant variability in cannabinoid profile and content compared to indoor cultivation methods. This results in difficulties in receiving reproducible material, which poses obstacles in terms of medicinal use purposes [112]. This is reflected in a study comparing cannabinoid content and its variability from 19 indoor and 11 in-field cultivations [113]. Summarized comparative results are shown in Table 4. Cultivation in the field was associated with higher THC, CBD, and CBN content, but also with their significant variability, reflected in the standard deviation values. Interestingly, CBG content was similar in both types of cultivation. It should be noted that only three varieties of Cannabis overlapped in both crops. However, the trend was similar to that shown in Table 4. In a study comparing three types of *Cannabis sativa* cultivated outdoors and indoors, the data were consistent in showing significantly higher levels of CBD in samples from outdoors compared to those obtained indoors [114]. Conversely, THC levels were lower, while CBG levels were significantly higher in outdoor samples [114].

The above data clearly show the influence of environmental parameters on the production of cannabinoids in plant material when grown in the ground. Their uncontrolled influence will act as a stressor, thereby increasing the production of metabolites. The achievement of high concentrations itself may be a desirable phenomenon, but the fact of not knowing what factor exactly influenced such a result, and thus the impossibility of obtaining reproducible material definitely is. Another downside of field cultivation can be seen in the THC:CBD ratio. For medicinal purposes, the ratio of 1:1 is widely used, which is much easier to achieve in a controlled, closed indoor environment [45].

Generally, through the cultivation process, there are a few essential environmental parameters that affect phytocannabinoid production, including light, water quantity and quality, air temperature and humidity, CO_2_, and fertilization.

#### 4.2.1. Light

Light is one of the key parameters that play a role in regulating the growth and development of *Cannabis sativa*, directly affecting the production of cannabinoids. The effect of light on these compounds can be considered from the perspectives of photoperiod, light spectrum, and intensity.

1. Photoperiod, or the length of the light period per day, is an important factor regulating the growth phases of hemp. Hemp is a short-day plant, which means that flowering is induced by shorter days (longer nights). Changing the photoperiod affects flowering time, which in turn affects cannabinoid accumulation. A study by Peterswald [115] showed that changing the photoperiod (14 h light:10 h dark) affected THC and CBD concentrations, with a marked increase in CBD.
2. Light spectrum refers to the range of wavelengths of light that a plant absorbs. Blue light (around 450 nm) is particularly important during the vegetative phase, promoting dense and compact plant growth. Red light (around 660 nm), on the other hand, plays a key role during the flowering phase, which can lead to increased cannabinoid production. Bilodeau et al. [116] in turn discovered that green light (520–560 nm) inhibits THC production. Proper balancing of the light spectrum, especially the use of full-spectrum light, can significantly affect the THC and CBD content of cannabis. Danziger and Bernstein [117,118] evaluated the effects of different light spectra on three cannabis strains. Using blue light versus red light (1:1 and 1:4) yielded better results than white LED light, increasing the levels of CBGA. In Managini et al.’s study, cannabis clones were grown under three types of lighting: HPS, AP673L (LED), and NS1 (LED) [119]. Differences in plant morphology and cannabinoid content were found, with higher CBG content under NS1 lighting and higher concentrations of CBD and THC under AP673L and NS1 compared to HPS. Moreover, studies showed that a light spectrum with high UVA levels also induced CBG accumulation [119]. Westmoreland studied the effect of blue photon fraction on cannabis yield and quality [120]. Increasing the blue photon fraction (from 4% to 20%) resulted in a decrease in flower dry weight yield but had no negative effect on CBD or THC concentration.
3. Higher light intensity usually increases photosynthesis, which can lead to higher biomass production and higher cannabinoid content [121]. A meta-analysis by Backer et al. [122] showed that light intensity increases CBD and THC content, supporting the theory that these cannabinoids are a defense mechanism against light stress. Similar results have been shown by McPartland [123]. Cannabis strains from India and Central Asia had higher THC levels compared to European strains.

#### 4.2.2. Water Quantity and Quality

Both water quantity and quality can affect cannabinoid production in cannabis. Stress caused by water deficits can lead to increased transpiration, leading to leaf wilting, and ultimately to necrosis and yield loss [108,124]. On the other hand, properly controlled water management can allow for increased production of secondary metabolites, including THC and CBD. Studies have shown that controlled drought stress at the late flowering stage can increase THC and CBD content in flowers by up to 67% compared to plants irrigated regularly without loss of floral biomass [125]. Park et al. found that a 7-day water deficit period increased CBG content, and slightly decreased CBD and THC content [126]. Calzolari et al. showed a positive correlation between CBD content and rainfall, suggesting that the amount of rainfall affects the percentage of CBD content in flowers [127].

Irrigating plants with high salt (NaCl) water can reduce biomass production but also affect phytocannabinoid composition. Formisano et al.’s study showed the response of cannabis to irrigation with salt supplied as NaCl solutions with electrical conductivity (EC) in the range of 2.0–6.0 mS/cm compared to a tap water control [128]. Irrigation with saline water significantly reduced biomass production at concentrations >4.0 mS/cm while enhancing CBD production.

#### 4.2.3. Air Temperature and Humidity

Temperatures have a complex effect on cannabinoid content [129]. High temperatures can reduce levels of some cannabinoids, such as CBG, but their effects on THC and CBD are less clear [126]. Humidity, on the other hand, especially its lower values, can increase THC content [130]. For example, in Paris et al.’s study, THC content was 30 times higher at lower humidity (50%) compared to higher humidity (80%) [131]. The proper management of temperature and humidity is key to optimizing cannabinoid production, but the effects can vary depending on the plant’s genotype and other environmental conditions [124].

#### 4.2.4. Fertilization

The most important nutrients for proper plant development are nitrogen (N), phosphorus (P), and potassium (K). An adequate supply of macro- and micronutrients is essential for the efficient and sustainable cultivation of any plant, including cannabis. The various nutrients not only affect a number of different biochemical processes taking place inside the plant but also affect the production of cannabinoids. Saloner and Bernstein evaluated the effect of N supply as an environmental factor on cannabinoid and terpene content in hemp plants [132]. They found that an increase in nitrogen supply resulted in a significant decrease in the concentration of two major cannabinoids, THCA and CBDA, by 69% and 63%, respectively.

Bernstein et al. analyzed the effects of adding various minerals, including humic acid, phosphorus, nitrogen, and potassium, onto the cannabinoid profile of cannabis grown on commercial irrigation media [133]. Each supplement affected cannabinoid concentrations differently depending on the plant organ. For example, NPK supplementation increased CBG concentrations in flowers by 71%, while CBN concentrations decreased by 38% in flowers and 36% in leaves. Interestingly, THC, CBD, and CBC concentrations remained generally unchanged with various K content [134].

Phosphorus supply at concentrations >5 mg/L reduced THCA and CBDA concentrations in inflorescences by about 25% [135]. On the other hand, total cannabinoid content per plant increased with a higher P supply, suggesting the need to adjust the phosphorus regime depending on the production goal. Other studies examined the effect of the ammonium-(NH_4_)-to-nitrate (NO_3_) ratio on plant function and the production of secondary metabolites, including cannabinoids [136]. An increase in NH_4_ supply led to a decrease in inflorescence yield and cannabinoid concentration. The best results were achieved with a dominant supply of NO_3_, where metabolite production and plant function were at their highest levels. A high proportion of NH_4_ (above 30%) induced toxicity and led to severe plant damage.

Massuela et al. compared the effects of two types of fertilizers (mineral and organic) at different dilutions on biomass, CBD yield, and nutrient use efficiency of N, P, and K [137]. Under nutrient stress, CBD concentrations increased despite lower inflorescence yields, maintaining a 95% CBD yield with one-third less fertilizer. Organic fertilizers showed lower utilization efficiency, suggesting the need to improve their bioavailability.

#### 4.2.5. CO_2_

An increase in carbon dioxide (CO_2_) concentration in the cannabis growing environment can have a significant impact on cannabinoid production, although these effects are complex and depend on many factors [129]. High CO_2_ concentrations increase the rate of CO_2_ assimilation and can accelerate plant growth, potentially increasing yield. Studies suggest that under ideal conditions, including high light intensity, increases in CO_2_ concentration can improve photosynthesis and plant growth, which, in theory, should translate into higher yields and quality [129]. However, higher CO_2_ assimilation rates do not always lead to increased cannabinoid yield, as the effects can vary depending on whether the products of photosynthesis are directed to flower development and phytocannabinoid production or to other parts of the plant, such as roots or leaves. Additionally, increases in CO_2_ concentration can affect the chemical profile of the plant, which can lead to changes in the content of secondary metabolites such as cannabinoids. Therefore, further research is needed to accurately assess the impact of CO_2_ on cannabinoid production and adjust cultivation conditions to optimize the quality and quantity of these compounds.

#### 4.2.6. Inflorescence Drying Method

Drying the inflorescence prevents the growth of microorganisms and enables long-term storage while maintaining potency, flavor, medicinal properties, and efficacy [106]. Maintaining a water activity level between 0.55 and 0.65 aw minimizes the risk of mold or fungal infection while maintaining flower quality [106]. Various drying techniques can be applied to achieve this goal. Comprehensive reviews were conducted in this field [106,138]. Air drying is the most conventional method, where whole plants or separate inflorescences are placed in the net or hung in the air in a dry, dark room at 18–25 °C and 45–55% humidity [139]. This method is time-consuming, preceded by manual or mechanical trimming. Slow drying promotes quality preservation but can also lead to a slow loss of terpenes and some cannabinoids. For example, during prolonged air drying, the cannabinoid content drops from 29% to 13% [139]

Another method involves rapid drying in an oven, vacuum chamber, or vacuum desiccator [138]. This process is much faster than air drying. High temperatures during oven drying can lead to cannabinoid degradation. For example, at 105 °C, THC content decreases, and CBN content increases [140]. Rapid drying can therefore negatively affect therapeutic potential.

Freeze drying, or freeze drying, involves removing water by sublimation in a vacuum chamber while maintaining very low temperatures [141]. It is an expensive and energy-intensive method. This method preserves volatile compounds and acidic forms of cannabinoids, which contributes to the high quality of the final [142]. Thus, the chemical structure and content of the cannabinoids are well preserved, unlike high-temperature methods, which can lead to their degradation.

The influence of the drying method on the cannabinoid content has been shown in studies by Chen et al. [143] and Challa [144]. Chen et al. studied various techniques, such as sublimation drying, air drying, and hot air drying [143]. They found that conventional drying provided CBD content ranging from 7.76 ± 0.03 g to 13.93 ± 0.03 g CBD/100 g dry weight but reduced CBDA decarboxylation. The implementation of hot air, on the other hand, increased CBDA conversion from 0.2%–14.1%, resulting in higher CBD content. Challa observed a marginal increase in CBD content with increasing drying temperatures, with the highest (2.783%) being in non-isothermal drying mode (40/70 °C at 25% humidity) and the lowest (1.276%) in a freeze-dried product [144]. These results clearly indicate that the method of drying affects both the total CBD content and the degree of CBDA decarboxylation, which can be optimized by appropriate choice of temperature and drying technique.

### 4.3. Post-Harvest Extraction

The extraction process makes it possible to obtain cannabinoid-containing product forms different from the dried flower. This includes extract forms such as oil, tinctures, full-spectrum high cannabinoid extracts, gels, crystallized CBD, THC concentrates, waxes, and many others [1,106,145]. Two main extraction methods can be distinguished: the solvent-free method and the solvent-based method. Solvent-free methods are considered more ecological and simpler but often result in lower mass yields and reduced concentrations compared to solvent-based techniques. A detailed review describing each method and its effect on cannabinoid content was prepared by Sainz Martinez et al. [145].

Different extraction methods have a significant impact on the content and quality of cannabinoids in the extracts obtained. Extraction with liquid-phase solvents (e.g., ethanol, hexane) can lead to extracts of low purity, requiring additional purification steps, increasing costs and production time. Distillation is effective in purification, however, causes thermal degradation of cannabinoids, altering their final chemical profile. Soxhlet extraction offers high yields but, like the solvent method, requires further purification. Ultrasound and microwaves aid the extraction process, increasing efficiency, but can also lead to the need for additional purification. The most advanced technique is supercritical CO_2_ extraction, which provides extracts with high purity, minimizing cannabinoid degradation. It is an environmentally friendly and long-term cost-effective method despite higher initial costs.

## 5. Conclusions

Phytocannabinoids, including THC, CBD, CBG, CBN, and CBC, present broad therapeutic potential in a wide range of physical and mental conditions. They have shown efficacy in treating chronic pain, reducing seizure activity, slowing neurodegenerative processes, psoriasis, acne, loss of appetite, sleep disorders, and psychosis. Dose dependence was notable in most cases, and thus, this requires careful management.

To optimize the therapeutic use of phytocannabinoids, cultivation practices and post-harvest processing must be carefully controlled. Indoor cultivation provides consistent cannabinoid profiles and ratios, which is crucial for therapeutic applications, while outdoor cultivation can achieve higher cannabinoid content, but with significant variability. Key environmental factors such as light, water, temperature, humidity, fertilization, and the drying method used affect cannabinoid content and must be carefully controlled, especially when a THC:CDB ratio of 1:1 is required. Broader data transfer is needed to determine what concentrations and ratios are effective in treatment to indicate to the producers which parameters should be manipulated to obtain the desired result.

## Figures and Tables

**Table 1:** The mechanism of action of THC.

Receptors or Canals	Mechanism of Action	Effects	References
CB1	Agonist	Psychoactive effect; Analgesic effect; Relieving muscle pain and spasticity; Increased appetite; Antiemetic effect; Protecting neurons from glutamate-induced damage.	[10,14]
CB2	Partial agonist (Although the latest research shows that it may have antagonistic effects)	Reduces inflammation; Improves neurological functions.	[15,16]
GPR55	Agonist	Reduces inflammation; Reduces neuropathic pain.	[17,18]
GPR18	Agonist	Analgesic effect; Reduces inflammation; Reduces intraocular pressure; Antihypertensive effect.	[19,20]
5HT3A	Antagonist	Antiemetic	[21]
PPARγ	Agonist	Decrease blood glucose levels; Vasorelaxation; Anti-tumor effects; Modulation of intestinal permeability.	[22]
TRP Channels (TRPV2, TRPA1)	Agonist	Anti-inflammatory; Analgesic.	[23,24]
TRPM8 Channel	Antagonist	Analgesic.	[25]

**Table 2:** Overview of CBD receptors, mechanisms, and associated effects.

Receptors or Canals	Mechanism of Action	Effects	References
CB1	Negative allosteric modulator	Modulate cognitive and emotional aspects of pain perception	[28,29]
CB2	Agonist or inverse agonist	Influence on pain processing and perception	[28,29]
GPR55	Inhibitor	Limits the tissue-injuring inflammatory responses	[28,30]
5-HT1A	Agonist	Anxiolytic and antiepileptic	[28,31]
α1-Adrenergic	Agonist	Antinociceptive	[8]
PPARγ	Activator	Decreases blood pressure; Reduces the severity of atherosclerosis; Increases the available nitric oxide	[8,28]
TRP Channels (TRPV1, TRPV2, TRPA1)	Activator	Anxiolytic, anti-hyperalgesic, and anti-inflammatory	[28,32]
TRPM8 Channel	Inhibitor	Anti-inflammatory	[28,33]

**Table 3:** Summary of cannabinoids used in treatment and their dosages for specific conditions. Can.—type of phytocannabinoid; Ref.—references.

Treated Symptom	Dose and Administration Method	Can.	Result	Ref.
Chronic pain in humans	The method of administration is through inhalation via the Syqe Inhaler. The medical device was designed to precisely aerosolize selective doses: 0.50 mg THC (0.537 ± 0.052 mg THC) or 1.00 mg THC (1.083 ± 0.076 mg THC).	∆^9^-THC	Both doses resulted in a notable reduction in pain intensity: 63.64% of the patients in the 0.5 mg dose and more than 69.57% of the patients in the 1.0 mg dose demonstrated at least a 2-point reduction in pain VAS score.	[52]
Chemotherapy-induced neuropathy (rats-based study)	Inhalation of vaporized cannabis plant material. Active cannabis contains 10.3% THC but negligible CBD (0.05%). Cannabis from each sample was vaporized using 460 mg of dry weight plant to achieve blood levels approx. 130 to 140 ng/mL.		Inhalation of THC-enriched cannabis suppressed paclitaxel-induced cold allodynia and chemotherapy-induced neuropathic nociception.	[53]
Fibromyalgia	Cannabis oil oral intake (THC: 24.44 mg/mL, CBD: 0.51 mg/mL). The initial dose was one drop (~1.22 mg of THC and 0.02 mg of CBD). The mean dose at postintervention evaluation was 3.6 drops of cannabis oil (~4.4 mg of THC and 0.08 mg of CBD).		An increase in quality of life in the cannabis group participants resulted in better well-being and more energy for the activities of daily living. Reduction in the frequency and intensity of pain attacks.	[42]
Epilepsy seizures (rats-based study)	Intraperitoneal injection (i.p.) of 30 mg THC per kg.		Complete termination of both behavioral and electrographic seizures in this refractory seizure model.	[55]
Treatment-resistant epilepsy in children	Oral administration of drops of CBD and THC-based oil (Berdolite, Bedrocan International, Veendam, Netherland). The maximum tolerated mean daily doses of THC were 0.3–0.4 mg/kg.		Across all patients, 65.7% had a ≥50% reduction in seizure frequency. 94.1% of those prescribed both CBD and Δ^9^-THC sustained a ≥50% reduction in seizure frequency, significantly higher than treatment without Δ^9^-THC (only CBD).	[39]
Chemotherapy-induced nausea and vomiting	Oral capsules containing THC:CBD mixture (2.5 mg:2.5 mg) three times per day.		Decrease in nausea and vomiting, but additional side effects appeared.	[45]
Appetite in patients with inflammatory bowel diseases	Inhalation of medical cannabis or oral administration of cannabis-based oil or a combination, depending on patient preference and clinical characteristics. The monthly dose of prescribed inflorescence was 25.7 ± 8.1 g/month (resulting in 2.0 ± 1.5 g THC).		After 3 months, a third of the patients reported a significant increase in their appetite. The results were acknowledged in a modest increase in BMI of those patients after 6 months of therapy.	[44]
Sleep disturbances and anxiety	Oral supplementation in the form of one soft gel 30 min before bedtime for 4 weeks. The set of substances that showed effectiveness consisted of 0.35 mg THC and botanicals (75 mg each of hops oil and valerian oil).		The set of substances showed effectiveness and improved sleep quality (based on the PROMIS™ Sleep Disturbance SF 8A survey), as well as decreased anxiety levels (based on the PROMIS™ Anxiety 4a survey).	[67]
Chronic neuropathic pain (mice-based study)	Mice consumed 15 mL of gelatin containing 1 mg of CBD for 3 weeks. The CBD concentration in the blood serum of mice was determined to be in the range of 3–5 ng/mL.	CBD	CBD significantly relieved allodynia compared to control mice. This effect was apparent on the first pain test after drug-gelatin presentation (day 5) and was maintained throughout the 3 weeks of testing.	[98]
Persistent inflammatory pain (mice-based study)	CBD was administered intraperitoneally (50 mg/kg i.p.) and intrathecally (50 µg i.t.). Each method of drug administration was tested separately.		Both methods and doses were effective in alleviating the inflammatory pain model in mice induced by intraplanar injection of 20 µL CFA (complete Freund’s adjuvant) (1:4 in saline) into one hind paw of the mice.	[99]
Treatment-resistant seizures	Patients took orally highly purified CBD medicine (Epidiolex^®^ Greenwich Biosciences Inc, Varlsbad, Kalifornia, USA; Epidyolex^®^ GW Pharma (International) B.V Amersfoort, Netherlands, 100 mg/mL) in specific doses as an adjunct to antiepileptic treatment. Patients received double-blind treatment for 14 weeks, which included a 2-week titration period dose escalation, starting daily dose of 2.5 mg/kg/day) and a maintenance period of 12 weeks of stable dosing of 20 mg/kg/day or 10 mg/kg/day.		CBD was effective in reducing seizures in refractory epilepsy types across the study population. There seemed to be no consistent dose response between the two doses, both showing therapeutic potential.	[100]
Skeletal muscle fatigue	Study participants drank 60 mg CBD solubilized with 250 mL water or a placebo drink directly after exercise, which caused muscle fatigue.		Participants were tested after 24, 48, and 72 h. In the group taking CBD, significantly lower parameters of muscle damage were observed 72 h after training compared to placebo.	[71]
Neuroinflammation caused by β amyloid in rats’ model of Alzheimer’s disease (rats-based study)	Intraperitoneal injection of CBD 10 mg/kg for 15 days.		Administration of CBD almost completely rescued the integrity of pyramidal neurons, had a neuroprotective effect, and stimulated neurogenesis in the central nervous system in rats injected with human β amyloid.	[101]
in vitro study simulating the conditions that occur during acne	Exposing the test cells (human immortalized SZ95 sebocytes) to 10 μM CBD solution.		CBD effectively inhibited lipid synthesis induced by either arachidonic acid or the combination of linoleic acid and testosterone.	[82]
Liver function	Study participants took CBD orally in various formulations immediately after a mixed macronutrient meal for 5 days. Each administered serving contained 30 mg of CBD and was taken once a day.		Taking CBD after a meal resulted in a favorable insulin and triglyceride response. It decreased circulating insulin and triglyceride concentrations during the first 30 min following food ingestion compared to placebo.	[83]
Chemotherapy-induced taste alterations	The intervention group self-administered an oral dose twice a day (2 × 150 mg CBD, morning and evening), starting the day before chemotherapy and continuing for eight days in total in every cycle of chemotherapy. Patients were followed for three cycles of chemotherapy.	CBD	A sensory test was performed prior to the fourth cycle. Patients receiving CBD had a better ability to differentiate between strong and weak tastes after three cycles of chemotherapy compared with the control group.	[84]
Sleep quality	Participants were instructed to consume orally one liquid gel pill per day containing purified, hemp-derived CBD (50 mg of CBD, following their last meal, 1–1.5 h before bed). The supplementation period lasted 8 weeks.		Results showed that daily ingestion led to significantly improved perceived sleep quality compared to a placebo group.	[85]
Sleep disturbance	Participants were instructed to consume orally 1 capsule containing 15 mg CBD each day with food before going to sleep. Supplementation lasted for 4 weeks.		Results showed a self-reported reduction in the sleep disturbance over the course of 4 weeks (based on the PROMIS™ Sleep Disturbance SF 8A survey).	[86]
Anxiety	Study participants took 300 mg CBD orally in capsule form a 2.5 h public speaking test (intended to induce anxiety).		Results showed that CBD significantly decreased subjective anxiety compared to the placebo group during the post-speech phase of the Visual Analogue Mood Scale (VAMS) protocol.	[102]
Anxiety combined with social phobia	Study participants took 600 mg CBD in the form of oral gelatin capsules 1.5 h before the public speaking test.		Results showed that pretreatment with CBD significantly reduced anxiety, cognitive impairment, and discomfort in speech performance in participants with social anxiety disorder.	[48]
THC-induced paranoid symptoms in humans	Study participants took capsules containing 600 mg CBD orally 3.5 h before receiving 1.5 mg THC intravenously.		Results showed that pre-treatment with CBD inhibited paranoia induced by THC. CBD also decreased the proportion of participants who experienced clinically significant acute THC psychosis.	[103]
Psychotic symptoms of schizophrenia in humans with schizophrenia or schizophrenic form psychosis	After a minimum period of 3 antipsychotic-free days, patients took CBD orally for 4 weeks. The control group took amisulpride. The starting dose of CBD was 200 mg per day and increased stepwise by 200 mg per day to a daily dose of 200 mg four times daily (a total of 800 mg per day) within the first week. The respective treatment was maintained for another 3 weeks.		CBD exerts clinically relevant antipsychotic effects that are associated with marked tolerability and safety. Participants showed significant clinical improvement, assessed by the reduction in PANSS (Positive and Negative Syndrome Scale) total score. Results suggest that CBD is as effective as amisulpride.	[89]
Cisplatin-induced neuropathic pain (mice-based study)	After induction of neuropathy, mice received daily intraperitoneal injections of CBG (10 mg/kg (i.p.) for males and 15 mg/kg (i.p.) for females) for 14 days.	CBG	CBG reduced neuropathic pain in a mouse model of CIPN in male and female mice without the development of tolerance or need for dosing more than once a day.	[91]
Chemotherapy-induced cachexia (rats-based study)	An acute cachectic phenotype was induced in rats by 6 mg/kg intraperitoneal cisplatin. CBG (120 mg/kg) was dissolved in sesame seed oil and administered orally in 1 mL/kg dose volume twice a day. Feeding behavior, bodyweight, and locomotor activity were recorded for 72 h.	CBG	CBG modestly increased food intake, predominantly at 36–60 h, and robustly attenuated cisplatin-induced weight loss from 6.3% to 2.6% at 72 h.	[93]
Inflammation in normal human epidermal keratinocytes and human dermal fibroblasts (in vitro study)	UVA- and UVB-induced inflammation and chemical and bacteria-induced inflammation were tested. The test cells were exposed to the CBG solution. Half maximal inhibitory concentrations (IC50) were 14.7 nM in UVB-induced inflammation, 0.3 µM in UVA-induced inflammation, 48 nM in chemical-induced inflammation, and 0.0003 nM in bacteria-induced inflammation.		CBG inhibits pro-inflammatory cytokines release from several inflammatory inducers, such as UVA, UVB, chemicals and bacteria, in several instances more potently than CBD.	[80]
Hepatic sphingolipid deposition and metabolism in a rat model of insulin resistance (rats-based study)	Rats were fed the high-fat diet and 20% solution of sucrose for 6 weeks and received CBG (30 mg/kg) dissolved in sesame oil via intragastric administration during the final 14 days of the study.		Results showed that CBG treatment modulates the sphingolipid metabolism, potentially preventing the development of liver steatosis. Treating insulin-resistant obese animals with CBG may improve liver insulin sensitivity.	[95]
Parkinson’s disease in neuroblastoma cells (in vitro study)	The study focused on mitochondria dysfunction induced by 1-methyl-4-phenylpyridinium (MPP+) Retinoic acid-differentiated SHSY5Y neuro-blastoma cells were treated with a medium containing CBN at the concentration 10 or 20 µM.	CBN	Results showed that CBN counteracted the loss of cell viability caused by MPP+ and reduced expression of genes involved in mitophagy, which was increased by MPP+.	[96]

**Table 4:** Cumulative comparison of phytocannabinoid content in plant materials retrieved from 19 indoor cultivations and 11 in-field cultivations (based on data from Aizpurua-Olaizola et al. [113]). Average ± standard deviation. The sample size for indoor is 19, and for outdoor is 11.

	THC, mg/g	CBD, mg/g	CBG, mg/g	CBN, mg/g
Indoor average	2.54 ± 1.0	2.60 ± 0.9	0.51 ± 0.3	9.39 ± 3.9
Outdoor average	12.24 ± 6.8	7.79 ± 3.5	0.48 ± 0.3	70.10 ± 13.6 *
Indoor minimum	1.10 ± 0.1	0.67 ± 0.1	0.067 ± 0.005	2.40 ± 0.1
Outdoor minimum	5.00 ± 0.1	2.20 ± 0.2	0.018 ± 0.001	5.90 ± 0.1
Indoor maximum	4.60 ± 0.3	4.50 ± 0.4	1.23 ± 0.1	18.00 ± 1.0
Outdoor maximum	25.00 ± 2.0	14.00 ± 1.0	1.17 ± 0.1	91.00 ± 6.0

* The sample size for CBN outdoor content was 10.

## Data Availability

The data availability statement is not applicable to this article.
